# Ultrasensitive and highly selective Co^2+^ detection based on the chiral optical activities of L-glutathione-modified gold nanoclusters

**DOI:** 10.3389/fchem.2024.1478021

**Published:** 2024-10-09

**Authors:** Qi Ding, Fang Wang, Weimin Yang, Xinhe Xing, Hengwei Lin, Liguang Xu, Si Li

**Affiliations:** ^1^ International Joint Research Center for Photo-responsive Molecules and Materials, School of Chemical and Material Engineering, Jiangnan University, Wuxi, China; ^2^ International Joint Research Laboratory for Biointerface and Biodetection, State Key Laboratory of Food Science and Technology, Jiangnan University, Wuxi, China

**Keywords:** chirality, fluorescence, nanoclusters, cobalt ion, detection

## Abstract

Developing highly sensitive and selective detection methods is crucial for environmental and healthcare monitoring. In this study, the chiral and fluorescent signals of L-glutathione-modified gold nanoclusters (L-GSH-Au NCs) were discovered to be responsive to Co^2+^, which displayed linear correlations with the concentration changes of Co^2+^. Notably, the chiral signal was more sensitive than the FL signal, whose limit of detection (LOD) was calculated to be 0.37 μM and 3.93 times lower than the LOD obtained with fluorescent signals. Moreover, the chiral signals exhibited unexpectedly high selectivity towards Co^2+^, effectively avoiding interference from other metal ions and biomolecules. Furthermore, the concentrations of Co^2+^ in various samples, such as Taihu water, tap water, bottled water, and animal serum, were accurately quantified using the chiral signals of L-GSH-Au NCs without complex pretreatment, with recoveries ranging between 95.64% and 103.22%. This study not only provides an innovative approach for Co^2+^ detection but also highlights the detection capabilities of chiral signals in complex environments.

## 1 Introduction

Cobalt ion (Co^2+^) is mainly present in the human body to form cobalamin (vitamin B12), playing a vital role in various catalytic reactions and myelin synthesis (Tvermoes et al., 2013; Kim et al., 2017). However, excessive intake can affect the nervous system and lead to heart failure and other diseases, such as pulmonary hypoplasia and thyroid damage (Lindsay and Kerr, 2011; Hussain et al., 2018; Saqib et al., 2018). Humans mainly take in Co^2+^ through food, water, and breathing. Therefore, it is essential to develop highly efficient and sensitive strategies to monitor the concentration of Co^2+^ in different propagation mediums, considering the health risks associated with Co^2+^.

Detecting metal ions typically involves traditional methods such as inductively coupled plasma mass spectrometry (ICP-MS) or atomic absorption spectrometry (AAS) (Hanhauser et al., 2020; Hou et al., 2020; Kong et al., 2020; Lin et al., 2020; Hong et al., 2021). However, these methods require extensive technical knowledge and time-consuming sample preparation. Despite being highly sensitive and accurate, they still need high requirements and tedious pretreatment (Shirani et al., 2019; Ma et al., 2020). To address these limitations, new detection methods have been developed using fluorescent spectrometry, electro-chemiluminescence, and colorimetry, which offer high convenience and rapid response (Xiao et al., 2016; Wu et al., 2023). However, with limited methods available, Co^2+^ detection with high sensitivity and selectivity still poses a challenge.

Chiral nanomaterials possess distinctive asymmetrical configurations, and their components can be manipulated, which allows for controlling chiral optical activities in the visible or near-infrared regions. This characteristic provides excellent anti-interference and high sensitivity towards configurational changes, making it possible to detect biological molecules such as disease markers, drugs, and metal ions with high sensitivity. For example, gold mimetic nanoparticles (L/D-P^+^ NPs) synthesized by Liguang Xu et al. possess excellent immunomodulatory capabilities and can be used to regulate the maturation of immune cells, in which the L-P^+^ NPs showed a higher (800-fold) efficiency as adjuvants for H_9_N_2_ influenza virus vaccination than commercial aluminum adjuvants (Xu et al., 2022). However, based on these advantages, using chiral nanomaterials for metal ion detection still needs to be explored.

**SCHEME 1:**
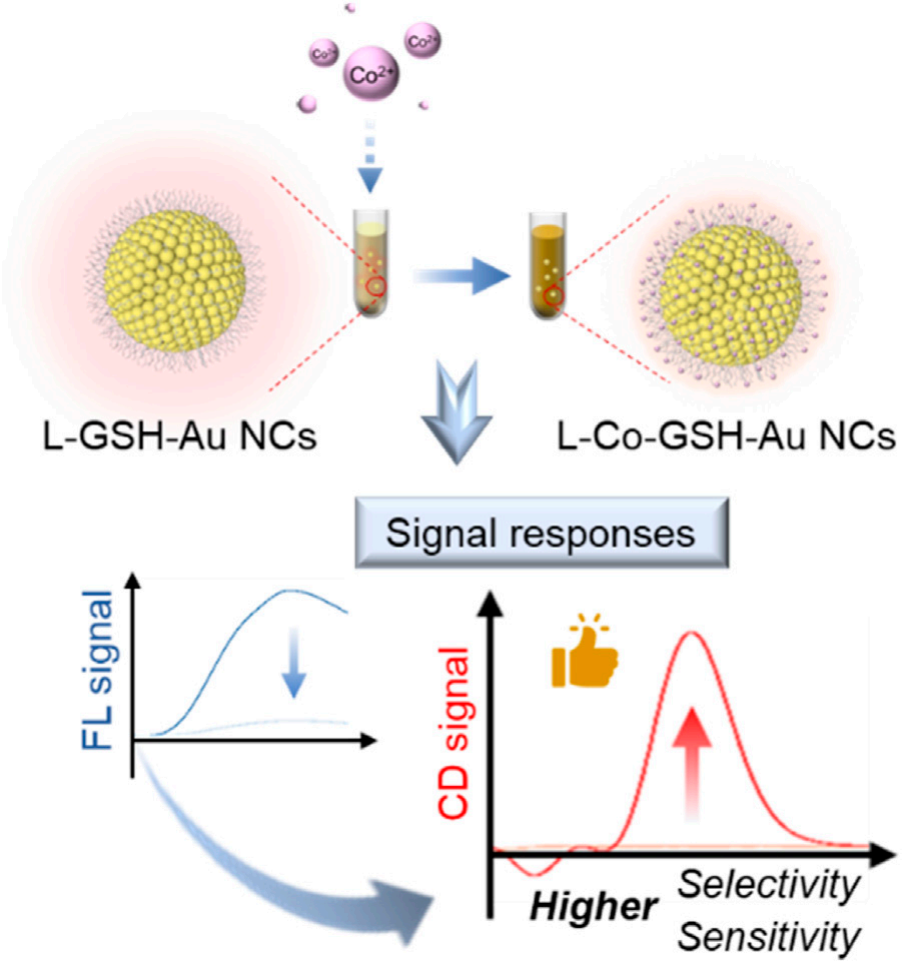
Scheme of Co^2+^ detection based on the chiral and fluorescent signals of L-GSH stabilized Au NCs.

Metal nanoclusters (NCs) are ultramicroscopic particles with specific optical properties, such as chirality and fluorescence (Zhang et al., 2020; Zhao and Li, 2020; Zhang et al., 2021; Sagadevan et al., 2022; Li et al., 2023). They have been used to construct metal ion-responsive luminescent probes based on aggregation-induced emission (AIE) characteristics, which displayed high sensitivity and signal-to-noise ratio. For example, Deyan Qi et al. synthesized CuAuNCs-Ce assemblies by exploiting the aggregation-induced properties between Ce^3+^ and glutathione-capped bimetallic copper and gold nanoclusters (CuAuNCs@GSH). The introduction of Ce^3+^ dramatically enhanced the fluorescence properties and quantum yield of the probe, which was applied to the highly sensitive detection of ATP with a detection limit of 53 nM (Qi et al., 2022). However, the chirality response of chiral metal NCs toward metal ions has yet to attract much attention. In this study, an innovative approach for Co^2+^ detection has been established based on the chiral responsiveness of L-GSH-Au NCs toward Co^2+^ (Scheme 1).

## 2 Experimental section

### 2.1 Regents and instruments

Gold chloride trihydrate (HAuCl_4_·H_2_O, 99%) was purchased from Sigma-Aldrich (Shanghai, China). Cobalt chloride (CoCl_2_, 99%) and glutathione (GSH, 99.9%) were purchased from Aladdin (Shanghai, China). The water used in the experiments was purified by the Milli-Q Biocel System, with a resistivity of 18.2 MΩ• cm. All chemicals were used as received without further purification.

Transmission electron microscopy (TEM) images were captured using a JEOL JEM-2100F transmission electron microscope (Hitachi, Tokyo, Japan) at an acceleration voltage of 200 kV. The circular dichroism (CD) signals were analyzed with a Chirascan plus CD spectrometer from Applied Photophysics (Surrey, United Kingdom) with an optical path length of 1 cm. Fluorescence spectra were obtained with a Hitachi F-7000 fluorescence spectrometer at room temperature. X-ray photoelectron spectroscopy (XPS) was performed using Axis Supra (Kratos, United Kingdom). Size distributions were determined with a Zetasizer (Malvern Instruments Ltd., Malvern, United Kingdom). The Shimadzu UV-vis 3101 spectroscope was used to obtain all ultraviolet light (UV)-visible absorption spectra. The Thermo-Nicolet Nexus 470FTIR spectrometer was used to perform Transform Infrared Spectrometer (FTIR) measurements. Fluorescence spectra were obtained with a Hitachi F-7000 fluorescence spectrometer at room temperature.

### 2.2 Preparation of L-GSH stabilized Au NCs (L-GSH-Au NCs)

L-GSH-Au NCs were synthesized using a thermal reduction method, as described in previous reports (Liu et al., 2016). To briefly summarize the process, 1.50 mL of 20 mM HAuCl_4_ was mixed with 8.56 mL 1.76 mg/mL L-GSH solution under stirring vigorously. The mixture was then heated in an oil bath at 95°C for 30 min to synthesize L-GSH-Au NCs. The mixed solution gradually changed from clear to light-yellow during the reaction. Once the reaction was complete, the synthesized L-GSH-Au NCs were purified through centrifugation at 11,000 rpm to remove any excess aggregates. Anhydrous ethanol was added to further purify the supernatant, which was then centrifuged at 3,500 rpm for 5 min. The remaining solution was resuspended in water, and the resulting L-GSH-Au NCs were stored at 4°C for later use.

### 2.3 Detection of Co^2+^

A gradient concentration of Co^2+^ solution (5 μL, range from 1 mM to 100 mM) was added into 500 μL as-synthesized L-GSH-Au NCs. The mixture was incubated at room temperature for 10 min. Afterward, the mixture’s CD, fluorescence, and UV absorbance signal were detected to determine the linear relationship between the concentration of Co^2+^, CD, fluorescence, and absorbance signals (Fluorescence excitation wavelength: 405 nm). Subsequently, the Co^2+^ concentration in each sample was evaluated based on the standard concentration curve.

### 2.4 Calculation and analysis of LOD

The LOD was calculated via sensitivity analysis. The calibration curve was presented as:

Y=a+bX

where a and b are variables obtained through least-squares linear regression of the signal–concentration curve, variable Y represents the C/fluorescence intensity of Au NCs at a Co^2+^ concentration of C (μM), and X is equal to log C.

The LOD was calculated as follows:

When b > 0,

$$Y=C_{\text{blank}}+3\times \text{SD}$$

$$\text{LOD}=10\times \frac{\left(C_{blank}+3\times \text{SD}\right)-a}{b}$$

where SD is the standard deviation and C_blank_ is the CD intensity or fluorescence intensity of the blank sample (without Co^2+^) and SD is the standard deviation.

When b < 0,

$$Y=C_{\text{blank}}\;–\;3\times \text{SD}$$

$$\text{LOD}=10\times \frac{\left(C_{blank}-3\times \text{SD}\right)-a}{b}$$

SD was calculated based on the formula:

$$\text{SD}=\sqrt{\frac{1}{N_{r}-1}\times \sum_{i=1}^{N_{r}}{\left(X_{i}-X_{avg}\right)}^{2}}$$

N_r_: total number of samples;X_i_: the CD intensity or fluorescence intensity of each sample;X_avg_: average value for the CD intensity or fluorescence intensity obtained for a specific series of identical samples repeated N_r_ times.

### 2.5 Analysis for real samples

Three water samples were collected to demonstrate the detection method’s applicability in environmental samples, which included lake water from Taihu Lake in Wuxi, China, bottled water from Wahaha^®^ purified water, and tap water from Jiangnan University, also in Wuxi, China. To remove impurities or insoluble substances, all water samples were pretreated with a 0.22 μm membrane filter (BRAND^®^) and then centrifuged at 9,000 rpm for 5 min to collect the supernatant solution. After that, Co^2+^ was spiked to the pretreated water samples and then detected with L-GSH-Au NCs (the final concentration of Co^2+^ was 10 μM).

The anticoagulant-treated mouse blood sample was centrifuged at 3,500 rpm for 5 min to separate the serum. Afterward, 1 mL of serum was diluted to 9 mL with ultrapure water and then treated with 1 mL HNO_3_ for 2 h. The pretreated serum sample was centrifuged at 4,000 rpm for 2 min to obtain the supernatant. Finally, Co^2+^ was spiked to the pretreated serum samples (the final concentrations of Co^2+^ are 10, 30, and 50 μM, respectively) and then detected with L-GSH-Au NCs. Animal experiments were carried out in accordance with the ethical guidelines of the Animal Welfare Committee of Jiangnan University. The experimental animal license used was SYXK (Su) 2016-0045. Animal Welfare and Ethical Review Number is JN. No20240630b1201231 [328].

$$\text{Recovery }\left(\%\right)=\frac{\text{amount}\;\text{in}\;\text{spike}\;\text{sample}-\text{amount}\;\text{in}\;\text{sample}}{\text{amount}\;\text{in}\;\text{spike}}\times 100$$

## 3 Results and discussion

**FIGURE 1:**
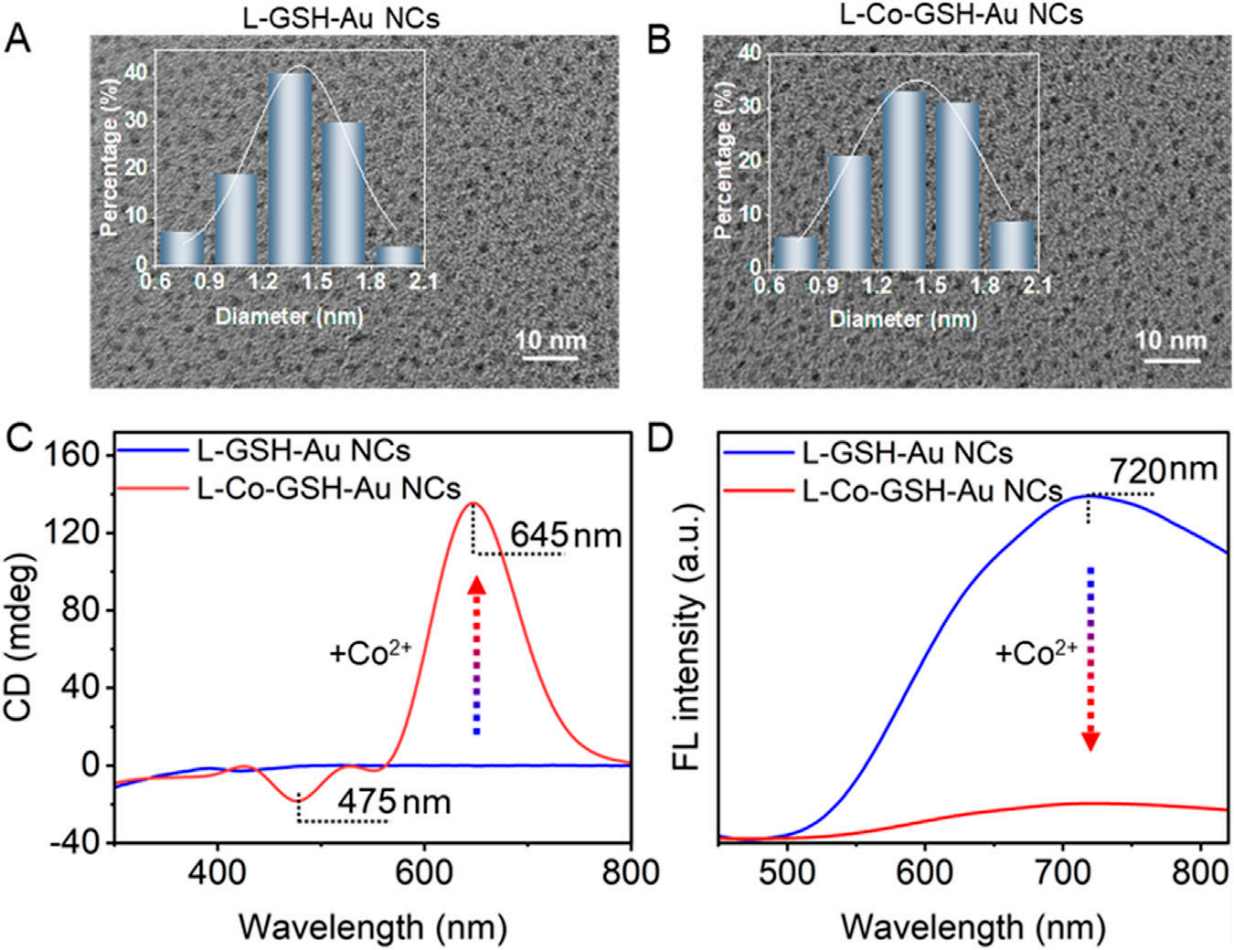
Transmission electron microscopy (TEM) images of **(A)** L-GSH-Au NCs and **(B)** L-Co-GSH-Au NCs (the insets show the size distribution histogram of L-GSH-Au NCs and L-Co-GSH-Au NCs). **(C)** Circular dichroism (CD) spectra and **(D)** fluorescence (FL) spectra of L-GSH-Au NCs and L-Co-GSH-Au NCs (Excitation wavelength: 405 nm). The final concentration of Co^2+^ is 500 μM for preparing the sample of L-Co-GSH-Au NCs.

Based on TEM images, the size of L-GSH-stabilized Au NCs (L-GSH-Au NCs) was determined to be 1.4 ± 0.3 nm (Figure 1A); the size of Co^2+^ treated L-GSH-stabilized Au NCs (L-Co-GSH-Au NCs) was analyzed to be 1.4 ± 0.2 nm (Figure 1B). After adding Co^2+^ with a final concentration of 500 μM, no noticeable morphological changes were observed in L-GSH-Au NCs. However, the CD and fluorescent (FL) optical activities of L-GSH-Au NCs changed obviously. In detail, the CD signal of L-GSH-Au NCs was initially distributed before 410 nm and shifted to the visible light region, with two new CD peaks appearing at 475 and 645 nm due to the addition of Co^2+^ (Figure 1C), accompanied by apparent changes in absorption (Supplementary Figure S1). The asymmetrical factor (g-factor) of L-GSH-Au NCs enhanced 40.22-fold after adding Co^2+^ (Supplementary Figure S2). Additionally, the FL emission peak of L-GSH-Au NCs displayed at 720 nm decreased significantly when Co^2+^ was added to the colloid solution of L-GSH-Au NCs (Figure 1D).

**FIGURE 2:**
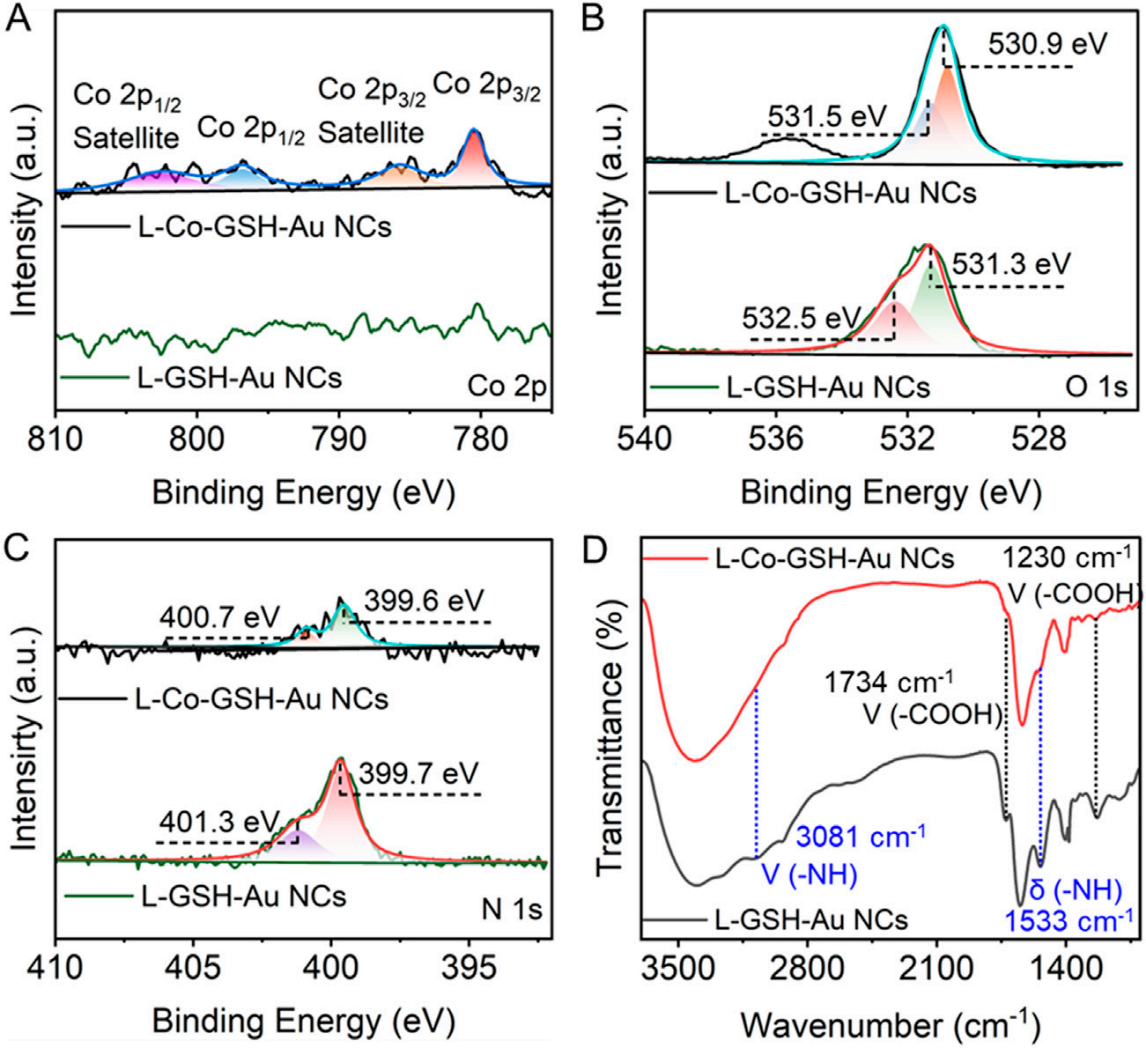
High-resolution X-ray photoelectron spectroscopy (XPS) spectra of **(A)** Co 2p, **(B)** O 1s, and **(C)** N 1s in L-GSH-Au NCs and L-Co-GSH-Au NCs. **(D)** The Fourier transform infrared spectroscopy of L-GSH-Au NCs (black), and L-Co-GSH-Au NCs (red).

The mechanism of the CD and FL signal changes of L-GSH-Au NCs induced by adding Co^2+^ was further investigated. First, no obvious morphological and size changes were observed after the addition of Co^2+^, thus the aggregation of L-GSH-Au NCs can be excluded (Figures 1A, B). Therefore, it is likely that a new coordination state was formed between Co^2+^ and individual L-GSH-Au NC. This deduction could be demonstrated by the decreasing surface charge when Co^2+^ was added to the colloid solution of L-GSH-Au NCs (Supplementary Figure S3). The measurement results of X-ray photoelectron spectroscopy (XPS) further illustrated the suspicion. They suggested the presence of Co elements in the sample of L-Co-GSH-Au NCs (Supplementary Figures S4, S5; Supplementary Table S1), which meant that Co^2+^ indeed interacted with L-GSH-Au NCs. The high-resolution XPS spectra of Co 2p that were distributed in the sample of L-Co-GSH-Au NCs displayed two binding energies at 780.5 and 796.9 eV, indicating that the Co ions were presented with +2 valence (Figure 2A). The binding energy of N and O also changed significantly due to the addition of Co^2+^, suggesting the probable coordination between L-GSH-Au NCs and Co^2+^ via the surface carboxyl and amino groups (Figures 2B, C). The Fourier transform infrared spectroscopy (FTIR) spectra of L-GSH-Au NCs and L-Co-GSH-Au NCs were also measured (Figure 2D). The FTIR peaks at 1,230 and 1734 cm^-1^ corresponded to C=O, and the FTIR at 1,533 and 3,081 cm^−1^ corresponded to -NH. These FTIR peaks decreased obviously due to the addition of Co^2+^, which proved that Co^2+^ indeed coordinated with the -NH and -COOH functional groups (Shi et al., 2022; Ding et al., 2024). The -NH and -COOH functional groups originated from the GSH molecules modified on the surface of L-GSH-Au NCs via Au-S covalent bonding. According to previous studies, Co^2+^ should coordinate with two GSH molecules on the surface of Au NCs in the specific mode of CoN_2_O_2_, which attributed to the signal change of chirality (Ding et al., 2024).

**FIGURE 3:**
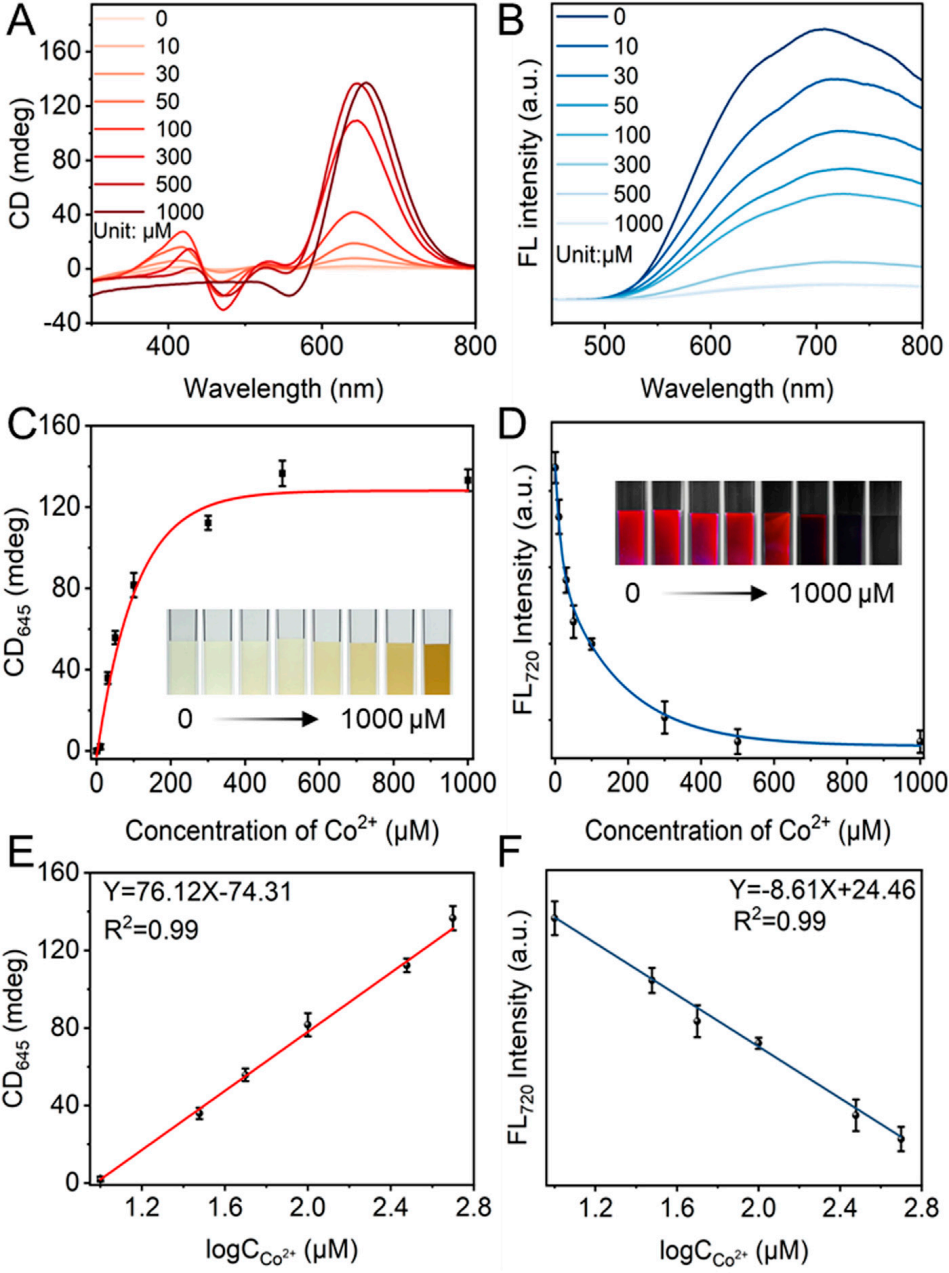
**(A)** Circular dichroism (CD) and **(B)** fluorescence (FL) of L-Co-GSH-Au NCs with the concentration of Co^2+^ changing from 0 to 1,000 μM (Excitation wavelength: 405 nm). **(C)** The CD intensity of L-Co-GSH-Au NCs at 645 nm and **(D)** the FL intensity of L-Co-GSH-Au NCs at 720 nm with Co^2+^ concentration ranging from 0 to 1,000 μM. The inset images in **(C, D)** are captured under daylight and UV light irradiation, respectively. Data are presented as mean ± standard deviation (n = 3). The linear relationships between **(E)** the CD intensity at 645 nm and the logarithm of Co^2+^ concentrations (logCco^2+^), and **(F)** the FL intensity at 720 nm and the logarithm of Co^2+^ concentrations. Data are presented as mean ± standard deviation (s.d.) (n = 3).

After that, the response-ability of the CD and FL signals of L-GSH-Au NCs was investigated by adding different concentrations of Co^2+^. The results showed that the CD intensity at 475 nm and 645 nm enhanced significantly as the concentration of Co^2+^ increased from 0 to 500 μM (Figure 3A), accompanied by the absorption increase and FL intensity decrease (Figure 3B; Supplementary Figure S6A). When the concentration of Co^2+^ was increased from 500 to 1,000 μM, the CD signal at 645 nm reached a plateau (Figure 3C). However, the absorption at 368 nm continued to increase (Supplementary Figure S6B). Additionally, the FL intensity at 720 nm slightly decreased, as depicted in Figure 3D. The negligible CD signal changes indicated that Co^2+^ occupied all the covalent binding sites on the surface of L-GSH-Au NCs. The absorption and FL signal changes with the concentration of Co^2+^ ranging from 500 to 1,000 μM were attributed to the aggregation of L-Co-GSH-Au NCs induced by the excess amount of Co^2+^ (Supplementary Figures S7, S8).

In order to test the detection sensitivity of the CD and FL signals, the linear relationships between the CD intensities and Co^2+^ concentrations, as well as the FL intensities and Co^2+^ concentrations were examined. It could be observed that the CD intensity at 645 nm and FL intensity at 720 nm showed good linear relationships with the logarithm of Co^2+^ concentrations ranging from 10–500 μM, respectively (Figures 3E, F). Meanwhile, the absorption intensity at 368 nm did not display any linear relationship with the concentration changes of Co^2+^ (Supplementary Figure S6C), which was attributed to the disturbance of the natural color of Co^2+^. The limits of detection (LOD) were calculated to be 0.37 μM and 1.45 μM based on the CD and FL signals. The results indicated that the CD signal was more sensitive than the FL signal under the same detection conditions. The higher detection sensitivity of CD signals should derive from their higher sensitivity toward the chiral configurational changes compared with that of FL signals.

**FIGURE 4:**
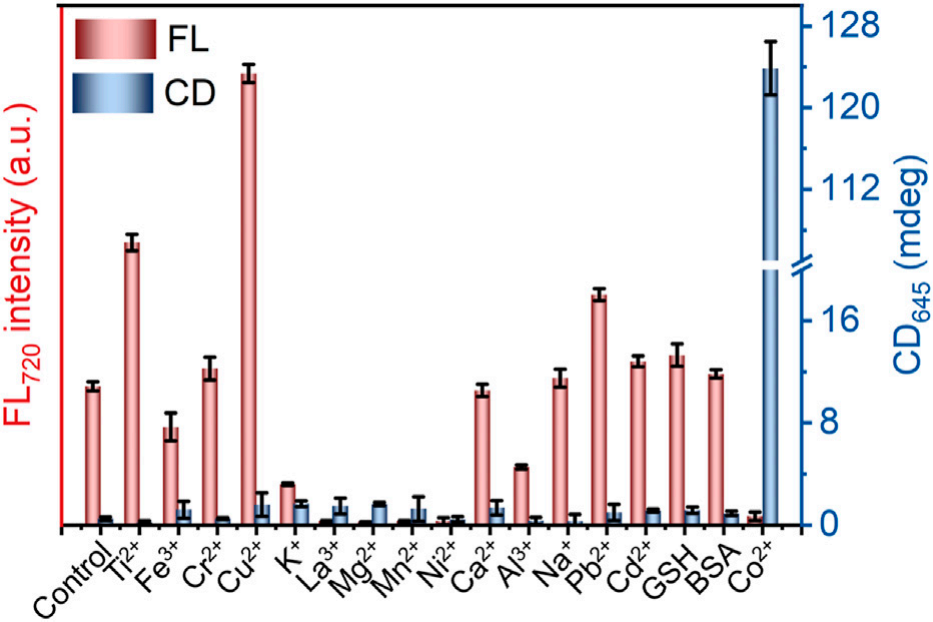
CD intensity at 645 nm and FL intensity at 720 nm of metal ions and biological interferences (500 μM) treated L-GSH-Au NCs, data are presented as mean ± s.d. (n = 3).

In addition to the detection sensitivity, the selectivity of the CD and FL signals was also studied. According to Figure 4 and Supplementary Figure S9, the FL intensity of L-GSH-Au NCs is enhanced in the presence of Ti^4+^, Cu^2+^ or Pb^2+^. On the other hand, GSH, BSA, Cr^3+^, Ca^2+^, Na^+^ and Cd^2+^ showed negligible effects on the FL intensity of L-GSH-Au NCs, while other metal ions resulted in decreases in FL intensity. However, among these metal ions and biological molecules, only Co^2+^ enhanced the CD signals of L-GSH-Au NCs (Figure 4; Supplementary Figure S10). These results illustrated that the CD signals of L-GSH-Au NCs exhibited high selectivity towards Co^2+^ while the FL signal did not. The high selectivity of L-GSH-Au NCs’ CD signal should originate from the specific coordination of CoN_2_O_2_ between Co^2+^ and L-GSH-Au NCs, which made the Co^2+^ differs from other metal ions.

Thanks to the high sensitivity and excellent selectivity of L-GSH-Au NCs’ chiral response, the Co^2+^ concentrations in various real samples, including Taihu water, animal serum, and tap water, were measured based on the CD signal of L-GSH-Au NCs. To investigate the detection ability of L-GSH-Au NCs’ chiral signal, the water samples were spiked with Co^2+^ to achieve a final concentration of 10.00 µM. As indicated in Table 1, the spiked recoveries of water samples ranged from 95.63% to 104.68%, with the relative standard deviations (RSDs) of 1.27%∼1.74% (n = 3). Moreover, the Co^2+^ concentration in the serum samples that were diluted 100-fold was spiked to be 10, 30, and 50 µM. The recoveries were detected to be ranging from 92.63% to 105.46%, with RSDs of 1.27%∼2.57%. Besides, the concentration of Co^2^⁺ in the real samples with spiked Co^2+^ was measured via ICP-MS. The Co^2^⁺ detection results from ICP-MS were consistent with those obtained from the CD assay, confirming that L-GSH-Au NCs can accurately detect Co^2^⁺ in real samples. Above all, L-GSH-Au NCs were proved to be a reliable and practical tool for detecting Co^2+^ in various samples.

**TABLE 1:** Determination of Co^2+^ in water and animal serum samples.

Sample	Spike (μM)	Found (μM)	Recovery (%)	RSD (%)^a^
Taihu lake water	10	9.69	96.94	1.74
		9.72	97.22	
		9.56	95.63	
Tap water	10	10.47	104.68	1.27
		10.25	102.53	
		10.11	101.07	
Bottled water	10	9.96	99.62	1.43
		9.99	98.63	
		9.63	96.26	
Human serum	10	9.81	98.13	1.65
		9.52	95.64	
		9.37	93.69	
Human serum	30	29.89	99.63	2.57
		27.79	92.63	
		28.88	96.27	
Human serum	50	52.73	105.46	1.72
		50.25	100.52	
		51.86	103.72	

^a^ RSD, relative standard deviation. All data were measured three times.

## 4 Conclusion

In conclusion, this study systematically investigated how L-GSH-Au NCs’ CD and FL signals respond when interacting with various metal ions and biological molecules. This research revealed that the CD signal of L-GSH-Au NCs not only displayed high sensitivity but also showed selectivity toward Co^2+^ compared to the FL signals. This unexceptional finding led to the successful development of a convenient method for detecting Co^2+^ without the need for complex pretreatment. Based on the chiral responsiveness of L-GSH-Au NCs, the detection method could accurately quantify the Co^2+^ concentrations in water and serum samples. This study demonstrated the detection potential of nanomaterials’ CD signals and introduced a new approach to developing highly sensitive and selective detection probes suitable for complex detection scenarios.

## Data Availability

The original contributions presented in the study are included in the article/Supplementary Material, further inquiries can be directed to the corresponding authors.
