# Supplementary material for: Ultrasensitive and highly selective Co2+ detection based on the chiral optical activities of L-glutathione-modified gold nanoclusters
Source: Front Chem. 2024 Oct 9;12:1478021. doi: 10.3389/fchem.2024.1478021 (PMC11496063; doi:10.3389/fchem.2024.1478021)

**Supporting Information**

**Ultrasensitive and highly selective Co^2+^ detection based on the chiral optical activities of L-glutathione-modified gold nanoclusters**

Qi Ding ^a^, Fang Wang ^a^, Weimin Yang ^a^, Xinhe Xing ^a^, Hengwei Lin ^a,*^, Liguang Xu ^b^, Si Li ^a,b*^

*^a^International Joint Research Center for Photo-responsive Molecules and Materials, School of Chemical and Material Engineering, Jiangnan University, Wuxi,* *214122, China*

*^b^International Joint Research Laboratory for Biointerface and Biodetection, State Key Laboratory of Food Science and Technology, Jiangnan University, Wuxi, 214122, China*


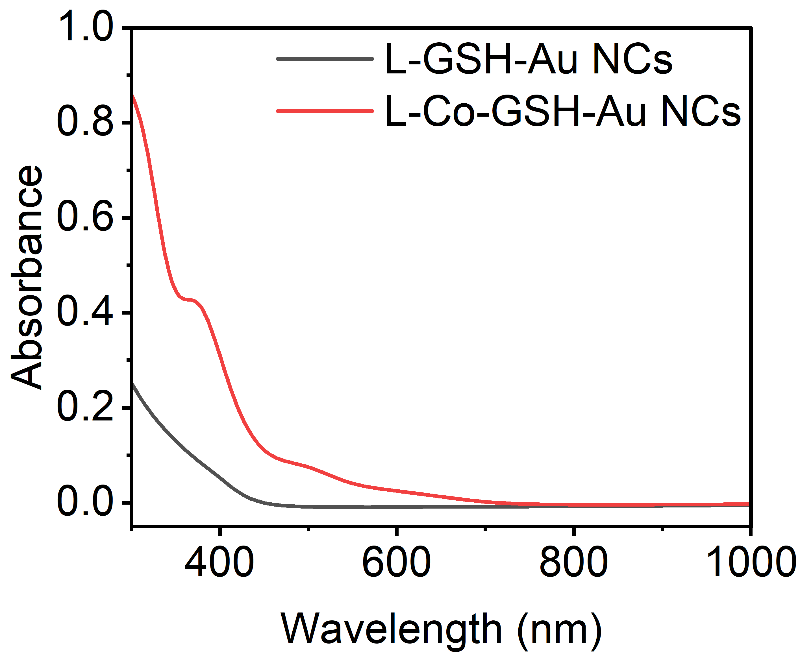


**Fig. S1.** The absorption spectrum of L-GSH-Au NCs and L-Co-GSH-Au NCs.


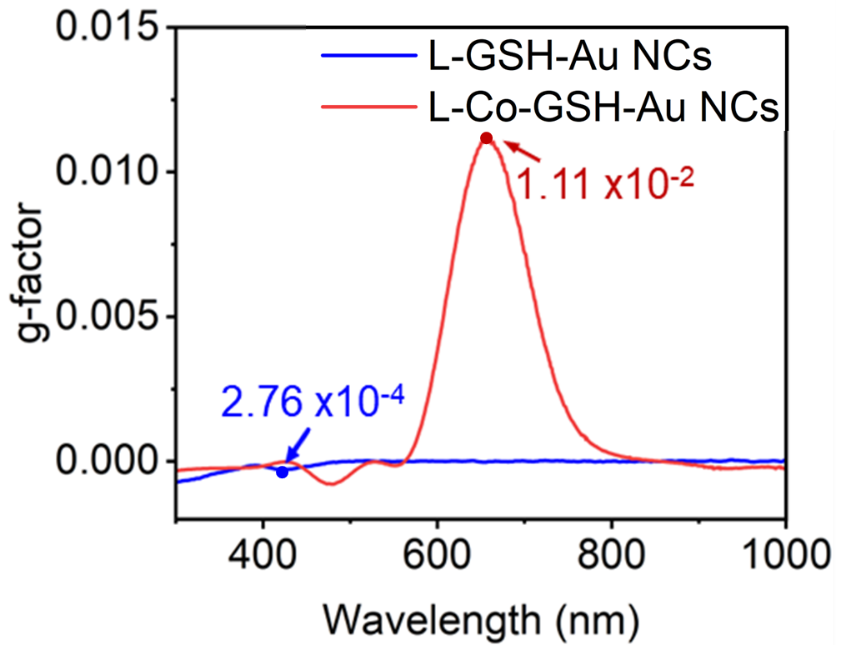


**Fig. S2.** G-factor of L-GSH-Au NCs and L-Co-GSH-Au NCs.


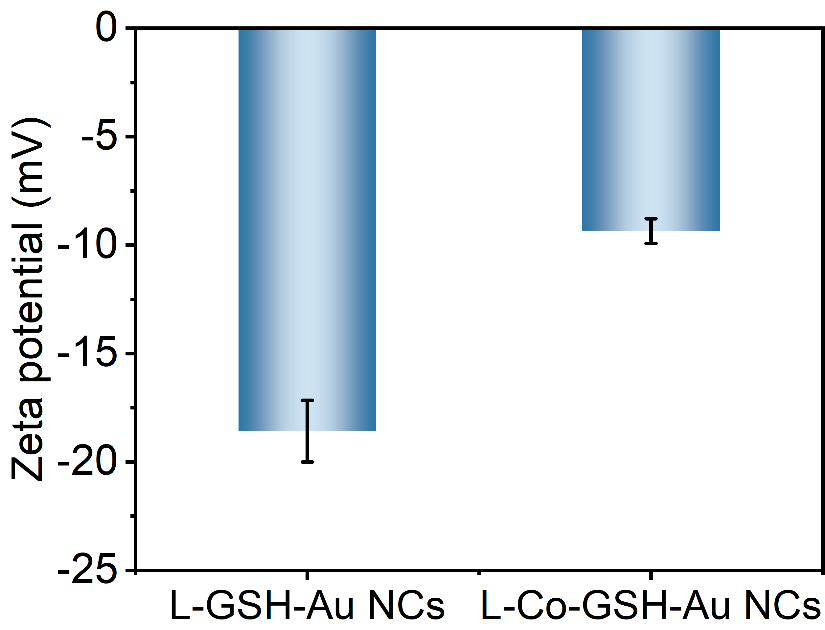


**Fig. S3.** Zeta potentials of L-GSH-Au NCs and L-Co-GSH-Au NCs.


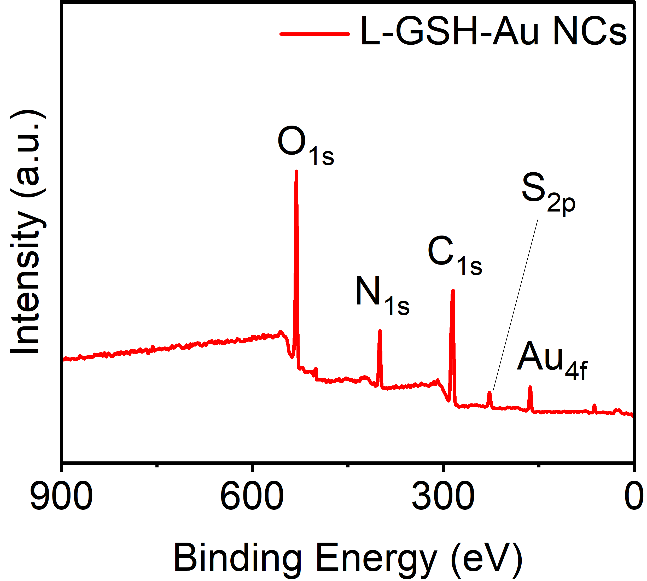


**Fig. S4.** X-ray photoelectron spectroscopy (XPS) survey spectrum of L-GSH-Au NCs.


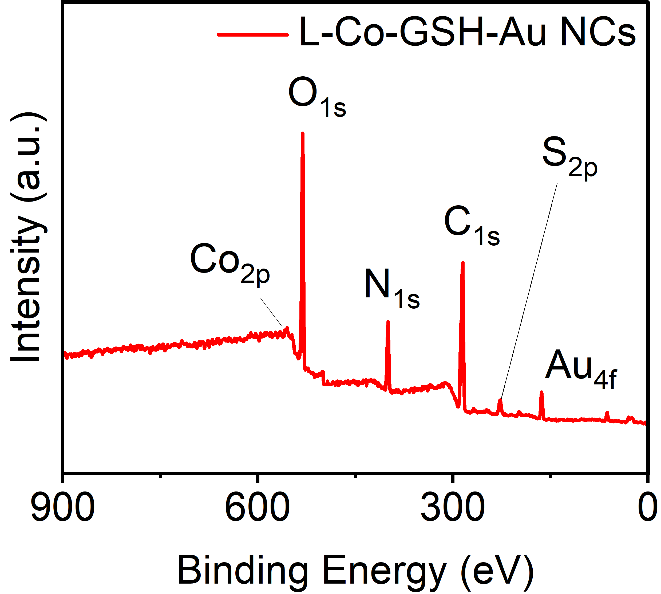


**Fig. S5.** X-ray photoelectron spectroscopy (XPS) survey spectrum of L-Co-GSH-Au NCs.

**Table S1.** Relative contents of C, N, and O in the L-GSH-Au NCs and L-Co-GSH-Au NCs determined by XPS.


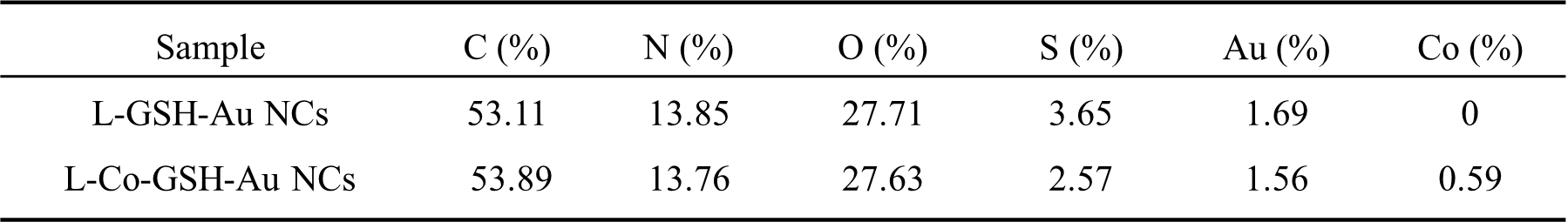


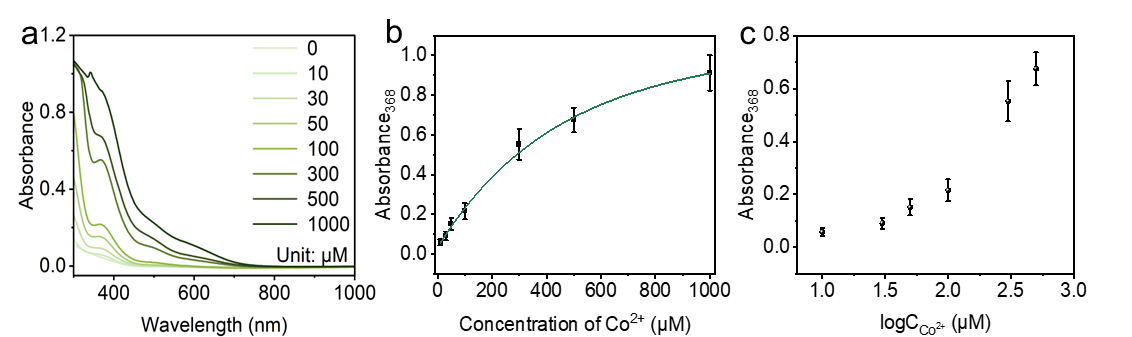


**Fig. S6.** (a) UV spectra of L-Co-GSH-Au NCs at different concentrations. (b) Absorbance intensity at 368 nm of L-GSH-Au NCs versus the concentration changes of Co^2+^. Data are presented as mean ± standard deviation (n = 3). The linear relationships between the (c) absorbance intensity at 368 nm and Co^2+^ concentrations. Data are presented as mean ± standard deviation (s.d.) (n = 3).


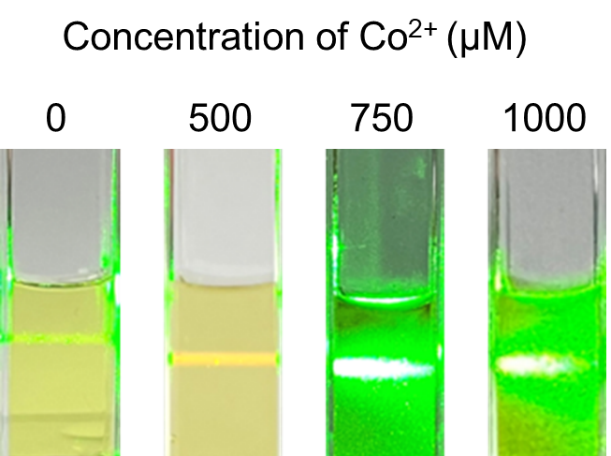


**Fig. S7.** Photographs of L-GSH-Au NCs treated with different concentrations of Co^2+^ (0, 500, 750, 1000 μM) under laser irradiation.


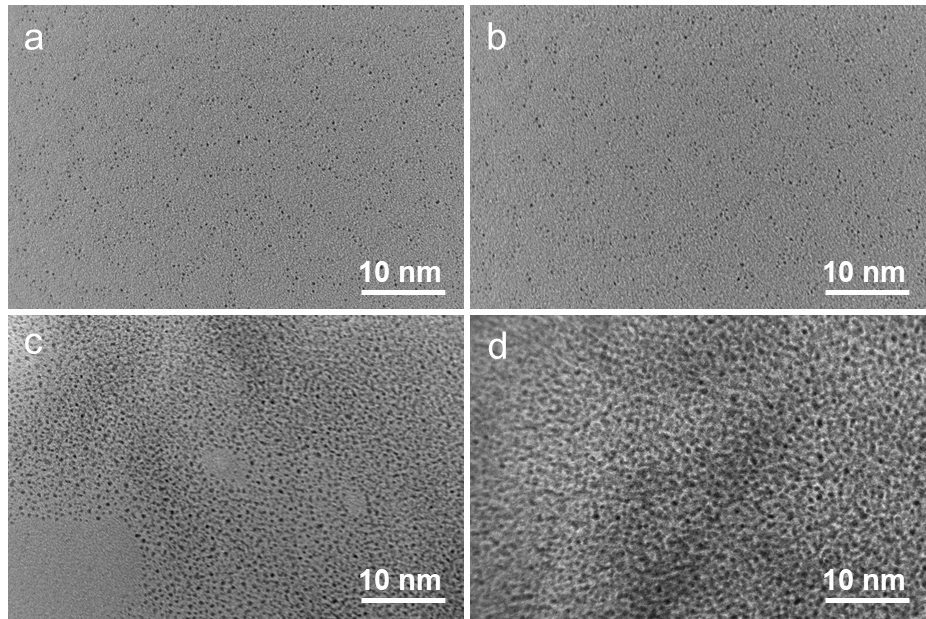


**Fig. S8.** Transmission electron microscopy (TEM) images of L-GSH-Au NCs treated with (a) 0 μM Co^2+^, (b) 500 μM Co^2+^, (c) 750 μM Co^2+^, (d) 1000 μM Co^2+^.


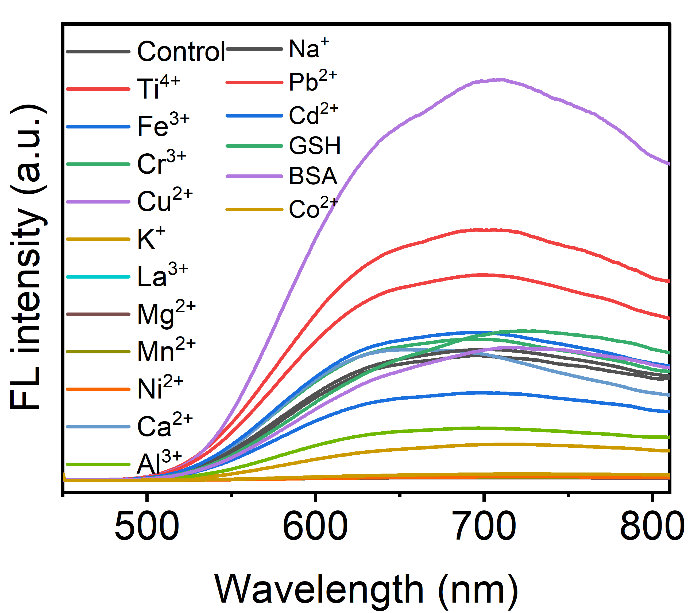


**Fig. S9.** Fluorescent responses of L-GSH-Au NCs toward different kinds of metal ions and biological interferences (500 μM).


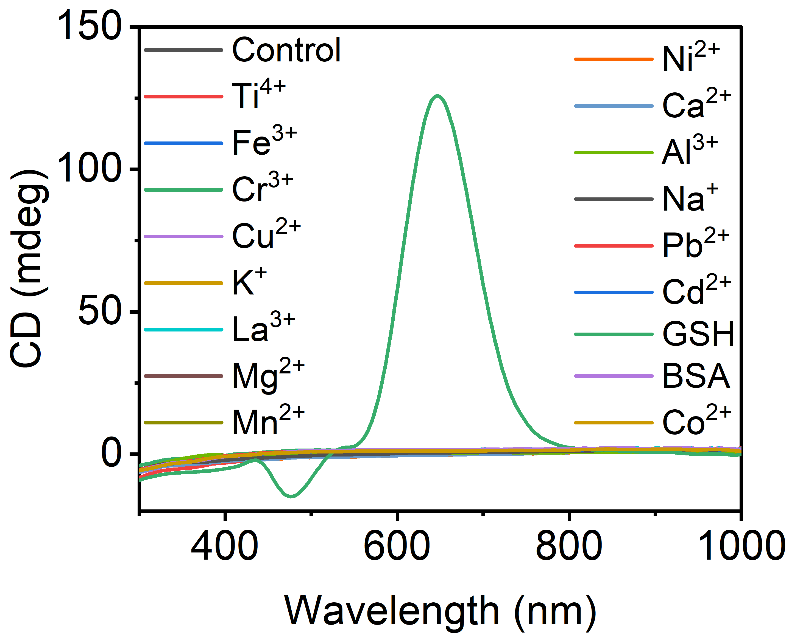


**Fig. S10.** Circular dichroism (CD) responses of L-GSH-Au NCs toward different kinds of metal ions and biological interferences (500 μM).

**Table S2.** Determination of Co^2+^ in water and animal serum samples via ICP-MS.


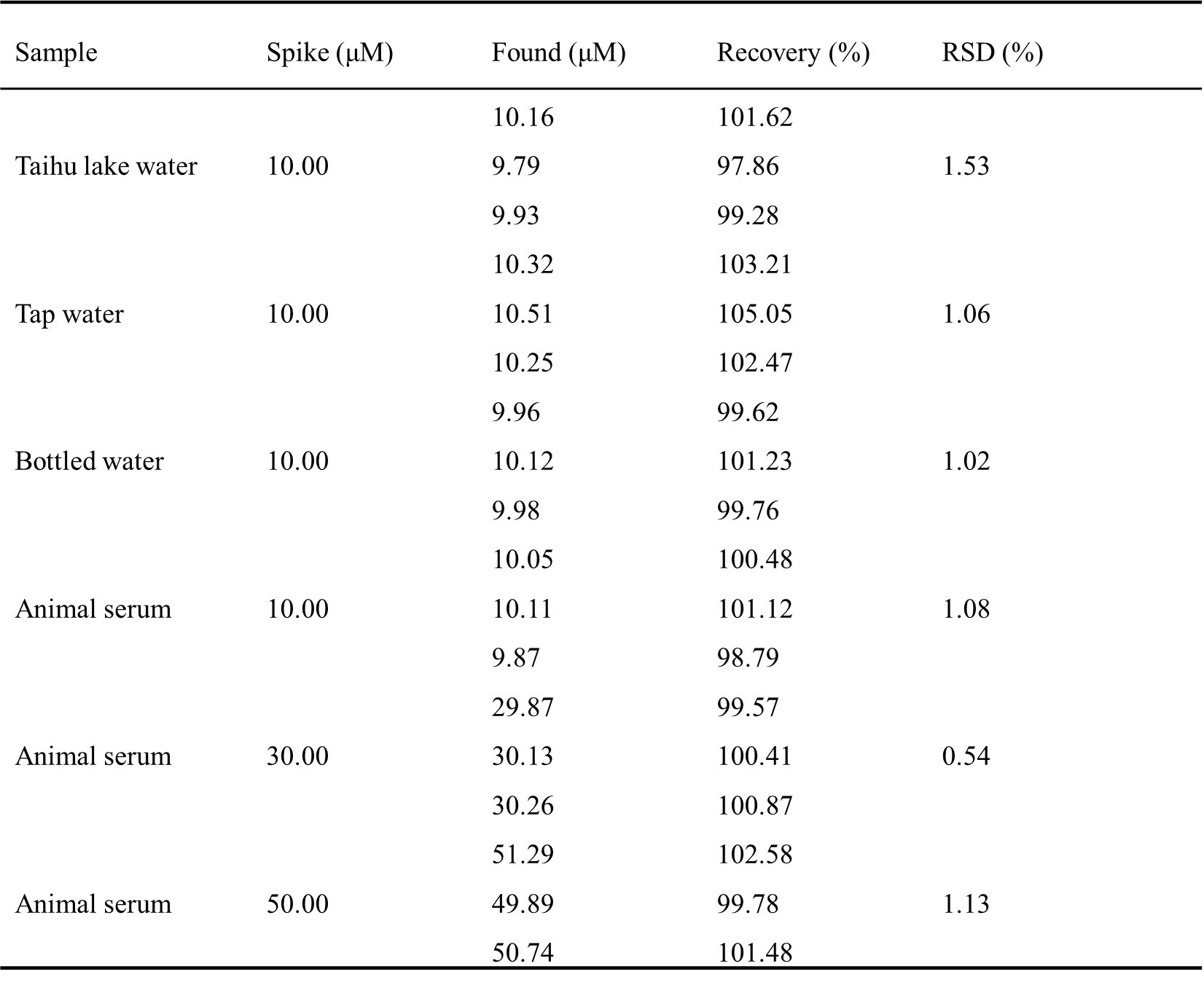

Supplement: Supplementary file 1 [file DataSheet1.docx]
